# The Medical World

**Published:** 1856-10

**Authors:** 


### EDITORIAL DEPARTMENT.

---

*The Medical World. A Journal of Universal Medical Science.*

We propose to welcome a new name in the catalogue of medical journals with a review, critical and analytical, of its merits. This is not our usual course—a simple word of greeting is our only notice on ordinary occasions. But this is not an ordinary occasion. It is not simply a journal of medical science, but a journal of *universal* medical science. It is not the child of some tyro ambitious of a name as a medical writer, but on its title-page it bears the name of J. V. C. Smith, M. D.—Alphabet Smith—as Editor—no less! J. V. C. Smith, for twenty-five years the editor of the Boston Medical and Surgical Journal, is now the editor of the Medical World, and its outer pages are covered with advertisements of this quack's Electro-Chemical Baths, and that quack's Treatment of Diseases of the Throat and Lungs by Medicated Inhalation, of Scientific Indian Physicians who cure cancers, of the New England Female Medical College, of Homœopathic Books and Medicines, of a "Lung Institute," of Cherry Pectoral and Oxygenated Bitters, of Balsam of Wild Cherry, and, to cap the climax of *Universal Medical Science*, a broad page devoted to setting forth the merits of "The Physiology of Marriage," a most remarkable book "for both sexes," a book to be kept hid in private drawers and read by stealth, and which "breathes, moreover, a truly Christian Spirit!!" We said once, that no man can be a thoroughly depraved and irredeemable quack until he has written a book on the physiology of Marriage. We take it back. The educated physician who advertises such a book has a right to all the titles of its author.

To thoroughly appreciate the position of the "Medical World," one must know something of the history of the Boston Medical Journal up to the time that Drs. Morland and Minot took charge of it, two years since, and made it an honorable journal.

Dr. Smith had been its editor for a quarter of a century. It was published at the American Athens, and ranked among its contributors the best names of New England, as well as some of the worst. For year after year the thing went on in such a way as no other journal ever did. The right of rejection of an article was one never exercised, (for occasionally a subscriber is lost in that way,) and side by side in the same number, were to be seen the brilliant productions of men of science, and the miserable self-puffing "report of a remarkable case" of some ignoble quack. All was grist that came to that mill. John C. Warren, and Dr. X. Chabert, of New York, were received with equal courtesy and each had honorable place.

So much for its contributors. In its department of reviews, it was most complaisant. From Rokitansky down to Mrs. Joel Shew, all were equally erudite, and every book sent to its editor was one "which should have a place in the library of every physician." Rarely did its editor presume to have opinions of his own, and when he did he merely hinted at their possession — did not assert them. For instance, some text-book of homœopathy is under notice: it is softly handled, and "commended to the kindly notice of those who, no doubt sincerely, advocate that mode of practice."

Aside from the queer mixture of excellence and quackery in its original department, and the uniform good-nature of its reviews, it had a character *sui generis* in its editorial pages. Nothing was attacked, everything was conciliated. The policy of the profession, we were told, was to soothe, not to irritate empiricism. And with what a gentle hand did this alphabetical editor rub down the limbs of quackery! How amiably he purred around homœopathy, and with what a truly Christian spirit of forgiveness did he look upon the sins of wate-cure! Legitimate medicine was right to be sure, but community would appreciate it better if it were not quite so right. It begged of the Massachusetts Medical Society not to expel its homœopathic members, it would hurt their feelings, excite unpleasant discussion, somebody would say something dreadful. The society yielded, and to this day the men of honorable scientific renown in that body, sit side by side with quacks at its meetings, in such a fraternal spirit as must warm the cockles of the heart of our editor. We can fancy his venerable figure leaning over them in the attitude of benediction, saying "God bless you, my children! How pleasant a thing it is to dwell together in unity!"

It is hardly to be supposed that everybody in Boston liked this sort of thing. Contributors of merit dropped off, and the Journal became the receptacle of more "remarkable cases" than any other was ever blessed with. The "solid men of Boston" laid their heads together, and called for some Curtius to save his city by going in and taking charge of the Journal. Drs. Morland and Minot, two men of talent, probity and learning, accepted the trust; and went in as assistant editors, though really having its entire control, except in the department of reviews. They made it what it should be, a good, honorable and valuable journal, having the interest of the profession at heart.

When we first saw their names as editors, nearly two years since, we wavered between rejoicing and regret. The Boston Journal had a large circulation, it was important that it should be ably edited, and therefore we rejoiced; but alas! we had been aching for years to plant a hearty kick or two at its former policy, and now the opportunity was lost, and *therefore* we mourned. Our grief overcame our rejoicing, and we were guilty of the indelicacy of welcoming Messrs. Morland and Minot to the editorial tripod with an ursine growl at their colleague. Our disappointment gave to our greeting — meant to be cordial — a sort of savage politeness. We said kind things of them, but expressed the ungraceful hope that they would make a better journal than their senior had done. Whereupon we "caught it" a little in return, which was all right — we deserved it.

Our history grows lengthy. When the journal had gone along for a while in a dignified, manly way, we must presume that Dr. Smith's sympathies did not harmonize, and accordingly we one day found his name missing from the title-page, and about the same time we heard of him in Kansas.

He has turned up again now. The opinions which have been budding for the last twenty-five years have now burst into full bloom in the "Medical World." It is a sort of *Quarter* Century Plant of Quackery.

We have known many medical men who have lived long lives in obedience to the dictates of legitimate medicine, who did so, not from any appreciation of its beauties, but because they were so stayed up by surrounding circumstances, that they were kept upright, *nolens volens*. If, with any of these men, a prop gives way, over they go headlong into quackery, and feel so much at home there that they wonder how they could have been such fools as to remain honest so long. The case now in hand seems to us something of this sort. In illustration we cut an occasional paragraph from our friend Smith's salutatory:

"Although educated to have entire confidence in the old school of medicine, as it has been taught in Europe and America by men illustrious for their attainments, long before many new and anomalous systems, which have their advocates and patrons, had existence, we believe also in the virtue of progress, and therefore open the pages of this journal to medical writers of every denomination throughout the United States.

"Our individual opinions shall not interfere with the freest expressions of those who differ from us on medical subjects."

"Old school!" To think that a man should edit a journal twenty-five years, and suppose all the time that it was the organ of a sect!

"Nothing, however, which reflects upon the personal character, professional standing, or influence of professors, practitioners, or authors, is admissible. Criticisms, however, on the writings, teachings, and theories of those representing the various theories at present taught in this or other countries, are legitimate methods of discussion."

That is just what we are doing now.

"Men differ on all subjects, and particularly so in religion, politics, and medicine, and they always will; but free discussion ought not to be suppressed by liberal-minded men in the practice of a liberal profession, on account of a jealousy that something may be lost that might have been kept by maintaining a dignified exclusiveness.

"The only feasible way of overcoming prejudice, and raising the medical profession, is to enlighten those ignorant pretenders, who bring disgrace upon it, by elevating them.

"Quacks abound, and quackery was never more rife, bolder, or more successful than at the present moment, a period distinguished for intelligence. Instead of quarrelling with the multitude, persuade them by all fair and honorable means to matriculate at the schools, listen to the instruction of public professors, and their ranks would soon present a different aspect.

"New schools, the very antipodes of our own, are perpetually on the increase, notwithstanding a spirited determination to keep them down. It is by that kind of persecution they thrive and multiply. However incompetent they may be to alleviate the physical disabilities of those who consult them, the masses sympathize with them, employ them because they are imagined to have been badly treated, and legislative bodies clothe them with collegiate powers upon the same principle. '*The blood of the martyr is the seed of the Church.*'

"With a philanthropic hope of gathering something from all these opposing elements, which may result in the fraternal advancement of the whole, without injury to any, we propose a new medical platform, based on mutual concessions, and a free, untramelled expression of sentiments.'

"The Medical World, with its broad open columns, is at the service of the whole medical public, by whatever name designated. Write and be friends."

What a mass of platitude is this! Has Dr. Smith forgotten, or did he never know, that the very essence of the creed of legitimate medicine is to have no sectarianism, to maintain its gateways ever open to improvement of any source, high or ignoble, and that a school, a sect, is necessarily narrow-minded. But we do n't mean to argue this case. It is beyond argument; and we leave it with the consolatory reflection, that the profession has lost nothing in respectability, in the withdrawal of a name which has always been tolerated, rather than honored.

